# Atrial fibrillation in retinal vascular occlusion disease and non-arteritic anterior ischemic optic neuropathy

**DOI:** 10.1371/journal.pone.0181766

**Published:** 2017-08-03

**Authors:** Josep Callizo, Nicolas Feltgen, Antje Ammermann, Janina Ganser, Sebastian Bemme, Thomas Bertelmann, Sebastian Pfeiffer, Andre Duvinage, Klaus Gröschel, Hans Hoerauf, Rolf Wachter

**Affiliations:** 1 Clinic for Ophthalmology, University of Göttingen, Göttingen, Germany; 2 Institute for Clinical Research GmbH, Georg-August University, Goettingen, Germany; 3 Clinic for Prevention, Rehabilitation and Sports Medicine, Technical University of Munich, Munich, Germany; 4 Department of Neurology, University Medical Centre of the Johannes Gutenberg University Mainz, Mainz, Germany; 5 Clinic for Cardiology and Pneumology, University of Göttingen, Göttingen, Germany; University Hospital Medical Centre, GERMANY

## Abstract

**Background:**

Patients with retinal vascular occlusion disease have an increased risk for ischemic stroke and share some risk factors with cerebrovascular disease. The purpose of this study was to analyze the prevalence of atrial fibrillation (AF) in subjects with retinal vascular occlusive disease and anterior ischemic optic neuropathy and to compare these data to an ischemic stroke group.

**Methods:**

Prospective, observational single-center trial. Subjects with retinal artery occlusion (RAO), retinal vein occlusion (RVO) and anterior ischemic optic neuropathy (AION) were included. Patients with ischemic stroke (IS) from a previous observational trial were used as control. Investigation included 7-day Holter ECG, echocardiography, duplex ultrasonography of the carotid arteries, and 24-hour blood pressure monitoring. Further vascular risk factors were documented.

**Results:**

During the 1-year study period, 101 patients were recruited. The control group with ischemic stroke consisted of 272 subjects. At inclusion, the prevalence of AF was 12% (RAO), 10.2% (RVO), 11.1% (NAION) and 15.8% (IS). The final prevalence after Holter ECG rose to 16% (RAO), 18.4% (RVO), 14.8% (NAION) and 26.5% (IS). No significant difference was measured between groups.

**Conclusions:**

We detected a similar prevalence of AF in all groups. RVO patients tended to exhibit a higher AF detection rate and lower number needed to screen than RAO and NAION. The detection of AF rose considerably via Holter ECG. As a consequence, we recommend prolonged ECG monitoring in patients with acute ophthalmic vascular diseases.

## Introduction

Acute retinal vascular occlusion, including arterial and venous occlusion, is the second most frequent retinal vascular disease worldwide.[1] Together with non-arteritic anterior ischemic optic neuropathy (NAION), they are among the most common causes of severe vision loss. Although the pathophysiology of these entities is not completely understood, patients exhibit a cardiovascular risk profile similar to that in ischemic heart and cerebral diseases[2,3].

Patients with retinal artery occlusion (RAO) and retinal vein occlusion (RVO) have a significantly increased risk for subsequent stroke and share similar risk factors (eg, atrial fibrillation (AF), carotid artery disease (CAD), hyperlipidemia, arterial hypertension, diabetes mellitus, smoking) with stroke patients.[4] Several studies have reported an association between RAO and stroke as well as acute myocardial infarction.[5–8] Among risk factors, AF is particularly relevant since it correlates with a five-fold increase in the stroke risk.[9] There is evidence that longer periods of monitoring can substantially increase AF’s detection.[10,11] It is now widely accepted that the prolonged monitoring of stroke patients by ECG identifies a significant number of patients with otherwise undetected paroxysmal AF.[12] Although retinal occlusion diseases share numerous risk factors with ischemic stroke (IS), no data on prolonged monitoring in either RAO or RVO patients exists. Screening for AF would therefore seem worthwhile. In a recent publication analyzing the cardiovascular profile in patients with central RAO, we found undiagnosed vascular risk factors in 78% of all patients[13]: 20% of the subjects presented AF. However, the role of isolated risk factors has not been analyzed in prospective studies. We hypothesized that, due to the similar vascular risk profile with stroke, patients with RAO and RVO may also reveal a higher prevalence of AF. Even though a vascular occlusion is presumed and several risk factors have been identified regarding NAION, there are few studies specifically addressing AF[14].

The main goal of this study was to analyze AF’s prevalence in subjects with retinal vascular occlusive disease and NAION and to compare these data to an ischemic stroke group’s.

## Material and methods

From June 2011 to June 2012, patients were prospectively recruited to participate in an observational single-center trial conducted at the University Eye Clinic Goettingen. Twenty-five patients had RAO, 49 had RVO, and NAION was diagnosed in 27 patients. The study protocol was implemented in accordance with the Helsinki Declaration. Ethics committee approval was obtained, and each patient provided written informed consent before participating in the study. All patients were admitted and underwent a standardized investigation including history, examination, blood tests, 12-lead 7-day Holter ECG (in patients without AF on admission), echocardiography, duplex ultrasonography of the carotid arteries, and 24-hour blood pressure monitoring. Risk factors such as stroke, TIA, ischemic heart disease, valvular heart disease, peripheral vascular disease, nicotine abuse, arterial hypertension, diabetes mellitus, and hyperlipidemia were documented. AF was defined as atrial arrhythmia lasting at least 30 seconds. CAD severity was assessed based on NASCET criteria. 272 patients with IS from a previous observational trial (Find-AF) with the same protocol were used for comparison[12].

Statistical analyses were done using SAS® 9.3 and the significance level alpha was set to .05. The relative frequencies of AF known at the time of inclusion and after the study (overall prevalence) were compared with chi-square test. The single comparison of the pooled data of RAO, RVO and NAION against IS was the primary contrast, so no alpha adjustment was needed. We also conducted pairwise comparisons against IS and between the groups RVO, RAO and NAION. To analyze risk factor profiles, we used descriptive statistics. For frequencies, the chi-square test or Fisher’s exact test and in case of continuous variables the t-Test were carried out for pairwise comparisons against the IS group (RAO, RVO and NAION alone and pooled). Continuous variables are expressed as mean ±SD whereas categorical variables are expressed as a percentage.

## Results

We recruited 101 patients during the 1-year study period. Of these, 25 (24.8%) had RAO, 49 (48.5%) RVO and 27 (26.7%) NAION. The study participants’ characteristics are shown in Table 1.

**Table 1:** Patient baseline characteristics. * single pairwise comparison of RAO, RVO or NAION against IS shows statistical significance (p<0.05). ** pooled data of RAO, RVO and NAION against IS shows statistical significance (p<0.05).

	RAO (n = 25)	RVO (n = 49)	NAION (n = 27)	IS (n = 272)
Age (years), mean ±SD	68.1 ±8.4	65.3 ±12.9 *	63.9 ±11.2 *	70.2 ±12.8 **
Gender, female	8 (32%)	19 (38.8%)	9 (33.3%)	121 (44.5%)
BMI, mean ±SD	27.3 ±3.8	28.8 ±6.3	28.5 ±4.8	27.7 ±5.7
Arterial hypertension	18 (72%)	28 (57.1%) *	11 (40.7%) *	201 (73.9%) **
History of atrial fibrillation	3 (12%)	5 (10.2%)	3 (11.1%)	43 (15.8%)
History of ischemic heart disease	7 (28%)	3 (6.1%)	5 (18.5%)	43 (15.8%)
History of peripheral artery disease	2 (8%)	1 (2%)	0 (0%)	8 (2.9%)
History of myocardial infarction	2 (8%)	3 (6.1%)	1 (3.7%)	20 (7.4%)
History of Stroke	1 (4%)	1 (2%) *	3 (11.1%)	42 (15.4%) **
Smoker	8 (32%)	11 (22.4%)	4 (14.8%)	57 (21.0%)
Diabetes Mellitus	2 (8%)	7 (14.3%)	8 (29.6%)	67 (24.6%)
Hyperlipidemia	7 (28%)	15 (30.6%)	5 (18.5%)	93 (34.2%)
Systolic pressure (mmHg), mean ±SD	157.5 ±31.7 *	146.6 ±26.1	146.4 ±23.8	144 ±25.0
Diastolic pressure (mmHg), mean ±SD	89.0 ±24.6 *	84.6 ±15.8 *	85.9 ±13.9 *	79.9 ±13.8 **

### Atrial fibrillation

**Fig 1:**
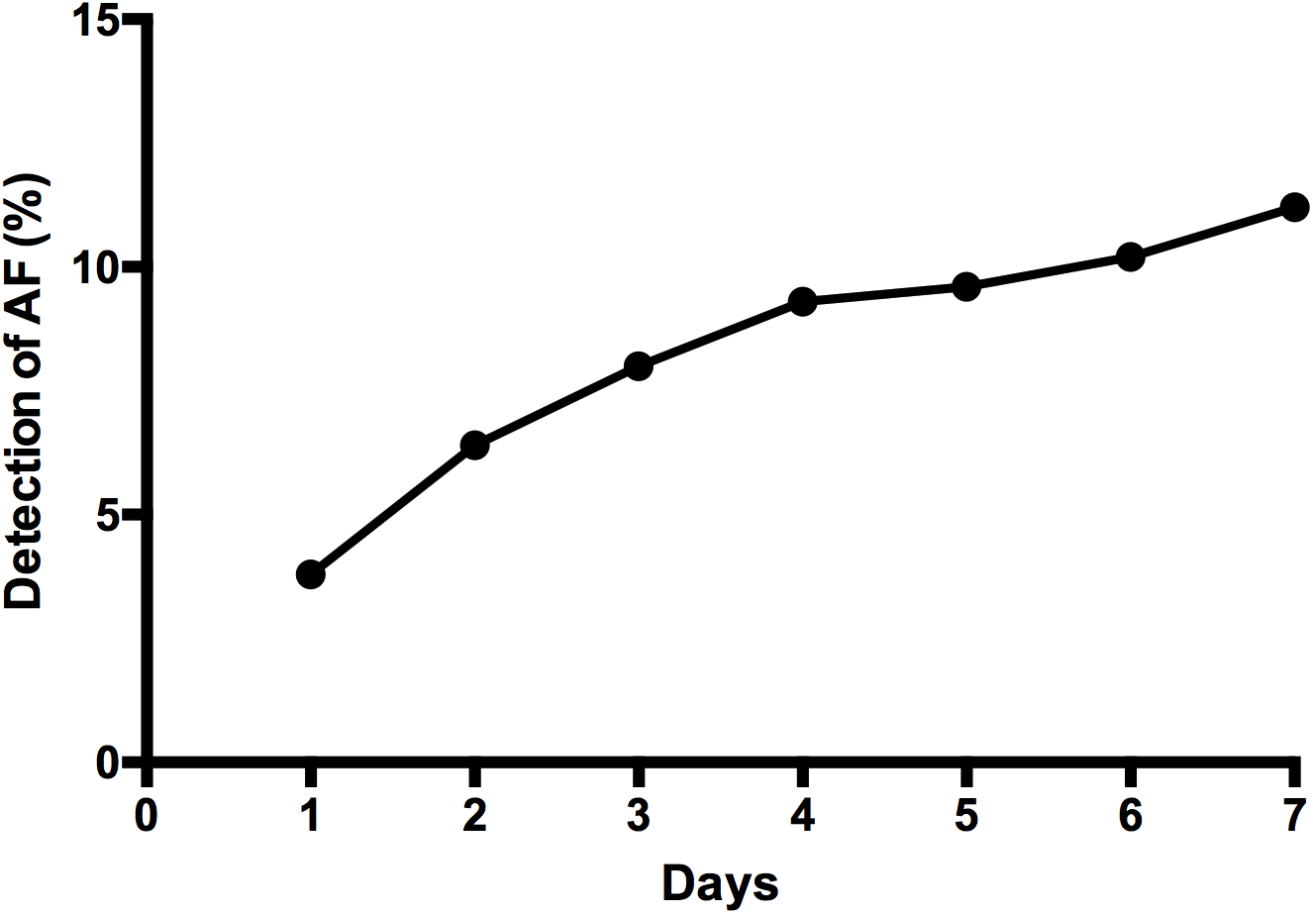
Detection rate of AF during the 7-day Holter ECG. Holter was perfomed among the 312 subjects (all groups) without previously known AF. On Day1, AF was detected in 3.8% of cases. After Day 7 detection rate increased to 11.2%.

**Fig 2:**
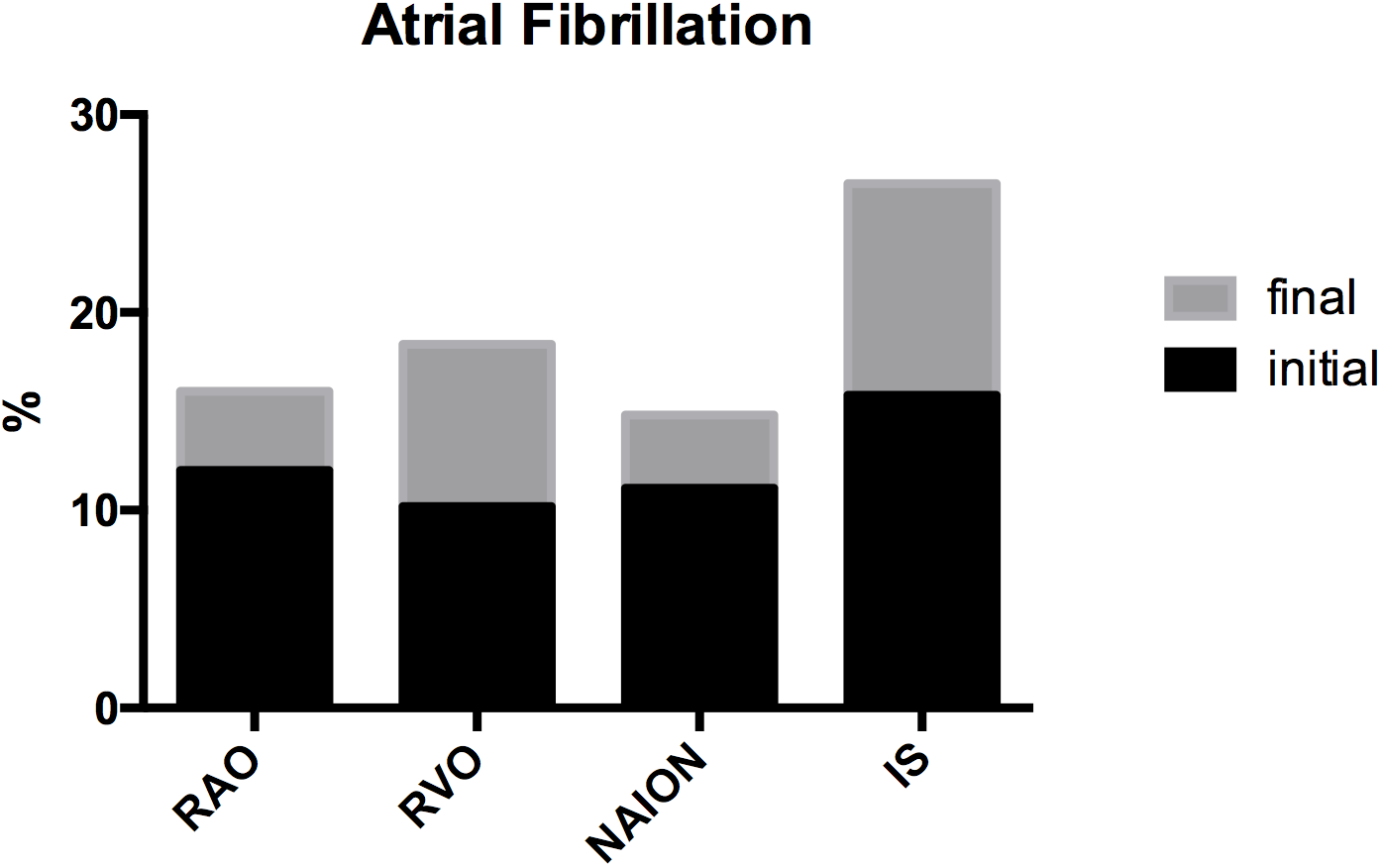
Prevalence of atrial fibrillation initial and after investigation with 7-day Holter ECG. Significant difference between groups was not observed.

At inclusion, the prevalence of known AF was 12% (RAO), 10.2% (RVO), 11.1% (NAION) and 15.8% (IS). No significant difference was measured between groups. No subject in the RAO, RVO or NAION group was newly diagnosed with AF on admission ECG. During the study, 312 (97.8%) patients from all 4 groups without known AF underwent heart-rhythm monitoring via a 7-day Holter ECG. Atrial fibrillation was detected in a total of 35 of them (1 RAO, 4 RVO, 1 NAION, 29 IS). There was no recognizable detection-rate pattern at any given time point during the monitoring period (mean time of detection: 3 days; range: 1–7 days) (Fig 1). The final prevalence of AF after Holter ECG rose to 16% (RAO), 18.4% (RVO) and 14.8% (NAION) and was similar in the three ophthalmic groups (p>0.05). The final prevalence of AF after Holter ECG was 26.5% in the IS group (Fig 2). The single and pooled analysis of AF presence revealed no significant difference between IS and any of the other three groups. Number needed to screen (NNS) to detect AF in RAO and NAION was 21. NNS was 10.5 in RVO, similar to that in IS (7.9).

### Cardiovascular risk factors in AF patients

In RAO and NAION, AF was detected in patients with a high cardiovascular burden of 5 to 6 risk factors, including ischemic heart disease and history of myocardial infarction. On the other hand, all RVO patients with newly diagnosed AF presented three risk factors. To estimate the risk of stroke in patients with AF, we applied the CHA_2_DS_2_-VASc Score.[15] Oral anticoagulation is recommended for patients scoring 2 and above. All of our patients with newly detected AF had a score >2, thus oral anticoagulation was recommended.

### Carotid artery disease

**Fig 3:**
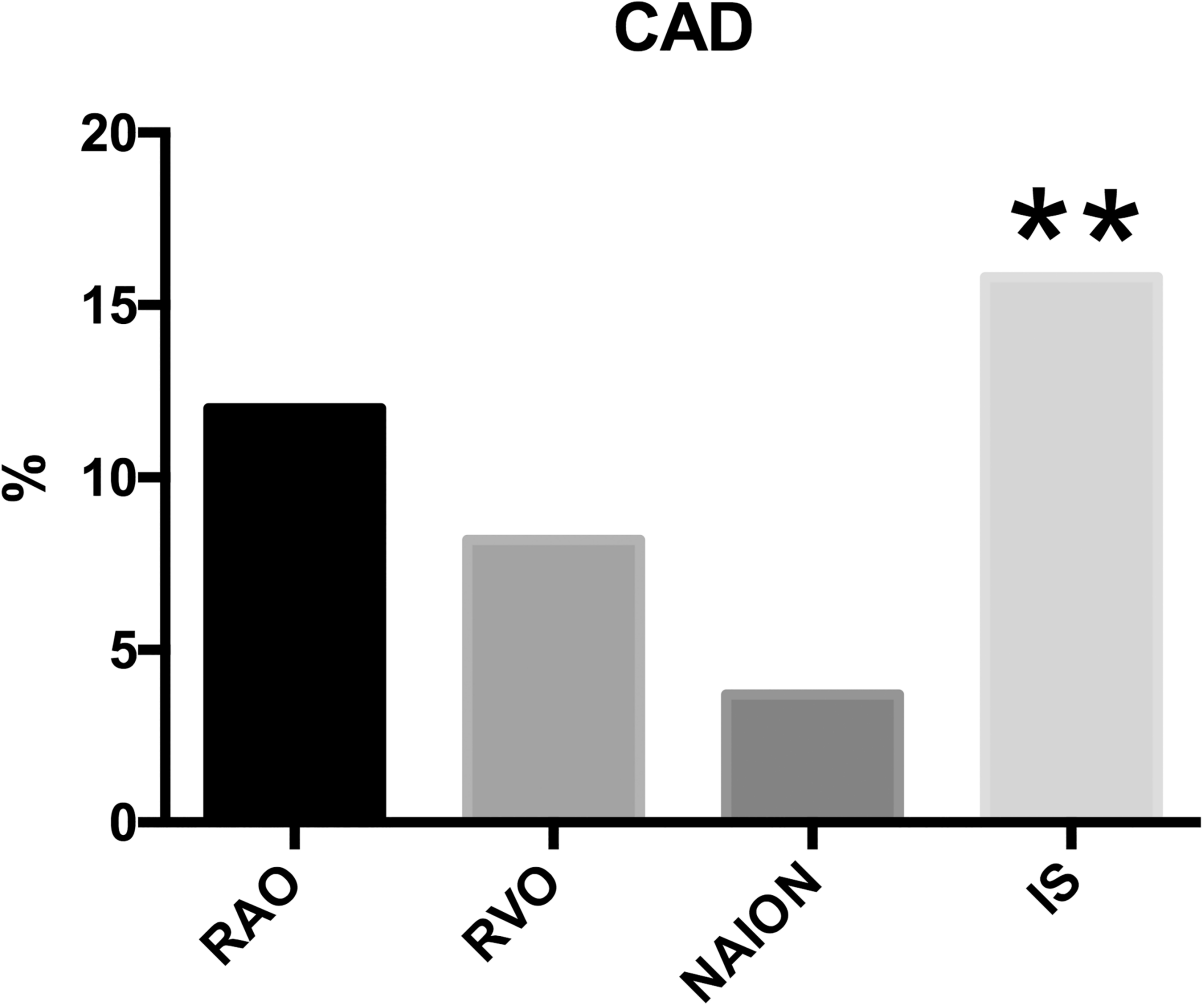
Prevalence of carotid artery disease after duplex ultrasonography of the carotid arteries. ** single pairwise and pooled comparison of RAO, RVO and NAION against IS shows statistical significance (p<0.05).

Among other risk factors, we also planned to screen for CAD due to its relevance. We carried out Duplex ultrasonography of the carotid arteries in 360 (96.5%) patients. A NASCET score of >50% (high-moderate to severe stenosis) was found in 12% of RAO, 8.2% of RVO, and in 3.7% of NAION patients (Fig 3). In comparison, 15.8% in the IS cohort had a score >50% in the IS cohort, which was significantly higher than the pooled prevalence in RAO, RVO and NAION (p = 0.04). However, we observed no difference in CAD between IS and RAO patients.

### Risk factors that significantly differ between groups

Patients in the RVO (p = 0.01) and NAION (p = 0.01) groups were younger than in the IS. The prevalence of hypertension in the RAO cohort (72%) resembled that in the IS (73.9%). However, it was significantly lower in RVO (57.1%, p = 0.02) and NAION (40.7%, p<0.01). Blood pressure values (especially diastolic) were higher in the eye cohorts. Systolic pressure was higher in the RAO (157.5 ±31.7) than in the other groups, particularly than in IS (144 ±25.0, p = 0.02). Diastolic blood pressure was significantly higher in RAO as well as in RVO and NAION as compared to IS. A history of previous stroke was more frequent in the IS cohort (15.4%). This difference became apparent in the pooled analysis (p<0.01) and, in the single analysis, concerning RVO individuals only (p = 0.01).

### Risk factors that did not differ between groups

Patients did not significantly differ in gender and BMI. The prevalence of history of ischemic heart disease, previous peripheral artery disease, previous myocardial infarction, smoking, diabetes and hyperlipidemia revealed no statistically significant difference between groups either. Although not significant, a previous ischemic heart disease diagnosis was more common among RAO patients (28%) than in the other groups. Similarly, previous peripheral artery disease, previous myocardial infarction, and smoking were also more prevalent among RAO patients, but this difference was not significant either. The percentage of diabetes and hyperlipidemia was highest in the NAION and the IS groups, respectively.

## Discussion

Our study’s main finding was the similar prevalence of AF in RAO (16.0%), RVO (18.4%), and NAION (14.8%) with no significant differences between groups. Furthermore, IS patients revealed the highest prevalence of AF and the lowest NNS (7.9). Their cardiovascular risk profile was very similar to those of RAO and NAION patients. Also remarkable was the fact that, despite being of similar or younger age, RVO patients tended to exhibit a higher AF detection rate and lower NNS than RAO and NAION. In RVO especially, AF’s detection via Holter ECG rose considerably (from 10% to 18.4%), reaching a higher level than in conjunction with arterial occlusions.

Recent studies have shown that after RAO and RVO, patients have an increased risk of ischemic stroke and myocardial infarction, particularly during the first weeks after the occlusion.[8,16,17]^,^[18] It is unclear whether this is an epiphenomenon explained by diseases sharing similar risk factors (eg, CAD is a strong risk factor for both IS and RAO, and atherosclerosis is a risk factor for myocardial infarction, ischemic stroke and RAO), or by sharing the same pathophysiology.

Retinal vascular occlusions are also a predictor for mortality. In a prospective population-based cohort study of 3,280 adults, Siantar et al investigated the relationship between eye diseases and mortality.[19] They found that only diabetic retinopathy and RVO were associated with increased cardiovascular mortality. Interestingly, RVO was a stronger predictor for mortality (hazard ratio 3.14; 95% CI, 1.26–7.73) than diabetic retinopathy (hazard ratio 1.57; 95% CI, 1.05–2.43). Tsaloumas et al followed up 549 patients with RVO over a mean 9 years[20] and demonstrated that death from acute myocardial infarction was significantly higher in RVO patients compared to other causes (23.1% versus 14.4%, p<0.05). Our results support the existing literature reporting a significant burden of vascular risk factors among these patients. In this respect, several population-based retrospective studies demonstrated that patients with retinal occlusion disease have a higher prevalence of cardiovascular risk factors than a control population.[3] A comprehensive, prompt diagnostic workup should thus be mandatory, since it enables us to identify a substantial number of patients with previously undiagnosed risk factors. In a previous study of ours, we demonstrated that 78% of RAO patients had undiagnosed vascular risk factors.[13] Among them, AF is especially relevant since it raises the risk for stroke dramatically[21], with a reported five-fold higher risk[22]. Another risk factor is CAD, which, especially when symptomatic, magnifies significantly the risk of stroke[23] and acute coronary events[24].

AF’s pathophysiologic role, especially in its paroxysmal presentation, is poorly understood. In a single case report[25], paroxysmal AF has been causally linked to RAO in a patient without other risk factors for RAO, but no systematic analysis of a larger patient cohort exists. Detecting paroxysmal AF is challenging because episodes are often brief, unpredictable, and frequently asymptomatic or accompanied by unspecific symptoms. The thromboembolic risk is similar in paroxysmal and persistent AF[26], and even short episodes lasting a few minutes are associated with higher rates of ischemic events[27]. The reported prevalence of AF in patients with retinal occlusions is highly variable. In most of the published data, the actual diagnostic method is not mentioned. In their population-based study, Chang and co-workers found that RAO exacerbated the risk for acute coronary syndrome. AF had been diagnosed in 1.16%[28] of their cohort of RAO patients. A population-based cohort study to determine the prevalence of RVO in Germany demonstrated a significant association between atrial fibrillation and RVO (Odds ratio 6.51, 95% CI 3.18–13.32).[29] In that cohort, a history of AF was positive in 3.1% of all participants and in 15.2% of those with RVO. Similarly, in a retrospective case-control study including RVO, 12.6% of the patients presented a history of AF.[30] The European Society of Cardiology’s most recent guidelines recommend at least three days of continuous monitoring, although 9 to 16% of stroke patients may have paroxysmal AF detectable by Holter ECG[31], external cardiac monitors[10] or implantable loop recorders.[11] Extending the electrocardiographic monitoring duration from 24 hours to 30 days increased the proportion of patients diagnosed with AF from 4.38% to 15.2%, suggesting a monitoring strategy longer than 24 hours is associated with higher detection rates of AF.[32] After 7 days of Holter ECG monitoring, we identified an AF prevalence of 16% (RAO), 18.4% (RVO) and 14.8% (NAION), reflecting a rise in the diagnosis of AF among RVO patients in particular (from 10.2% to 18.4%). Anderson et al calculated the odds for hemispheric and retinal ischemia in patients with AF and medically treated carotid stenosis[33], finding that retinal ischemia was more often associated with a carotid stenosis than with AF. This observation resembles that in a previous study of ours.[13] However, in the present study we detected a higher prevalence of AF than CAD after a prospective analysis in both arterial and venous occlusions. In an analysis of patients with ischemic stroke, TIA, RAO, and amaurosis fugax, Mead et al found that AF was more common in cerebral than ocular events.[34] In our study, AF was also more common in IS than in the ophthalmic groups, but the difference was not statistically significant. In a recent publication, Hayreh and Zimmermann report a worse carotid artery stenosis in patients with amaurosis fugax and ocular ischemic syndrome compared to retinal artery occlusion and NAION[35].

Taking an approach we share, Plunkett et al emphasized the importance of carefully screening for AF among RAO and RVO patients.[36] They advocate that if AF is present, a serious risk of stroke and systemic thromboembolism will persist if such patients are not anticoagulated. Christiansen et al published data supporting this opinion.[4] They were able to show that retinal vascular occlusions, either RAO or RVO, are independent predictors of stroke and systemic thromboembolism in patients with AF. Similarly, Park et al analyzed the periods of higher risk for cardiovascular events after retinal occlusions and showed that after central RAO, patients suffer an increased risk of stroke, particularly during the first post-RAO week with an incidence-rate ratio of stroke/myocardial infarction of 44.51 (95% CI, 27.07–73.20).[8] Analogously, they observed an increased risk for stroke after RVO, particularly during the first 30 days after RVO with an incidence rate ratio of stroke/AMI of 2.66 (95% CI, 2.06–3.43).[16] There are few population-controlled studies on NAION and its risks. In a review publication, Biousse et al postulate that although ischemic optic neuropathies and cerebrovascular diseases share some risk factors, they represent two entities and do not require the same work-up.[37] However, the prevalence of AF and ischemic heart disease observed in our cohort was similar in all groups, with a previous stroke being more common in the NAION group. An elevated stroke risk after NAION has also been recently reported[38].

## Conclusions

Our study is the first to investigate the use of 7-day Holter ECG in RAO, RVO and NAION patients. Although AF’s pathophysiologic role in arterial occlusion can only be assumed, and whereas venous occlusion is not a cardio-embolic disease, the present data reveal greater AF detection via prolonged monitoring in all groups in a percentage similar to patients who have had an ischemic stroke. The number needed to screen to detect AF in the ophthalmic groups ranged between 10.5 and 21, in comparison to 7.9 in IS patients. As a consequence, based on Holter ECG’s noninvasiveness and proven cost-effectiveness, we recommend prolonged monitoring in patients presenting such acute ophthalmic vascular diseases. Furthermore, as all our patients with newly detected AF had a high estimated risk of stroke (and due to their CHA_2_DS_2_-VASc scores), we recommended oral anticoagulation.

The main limitations of this study are the low number of patients included in the ophthalmic groups as well as the use of a historic cohort as a comparison. Further prospective studies recruiting a larger number of patients are necessary to confirm the role of AF and other cardiovascular risk factors in patients with acute ophthalmic vascular diseases.
